# Effectiveness of 'motivational interviewing' on sick leave: a randomized controlled trial in a social insurance setting

**DOI:** 10.5271/sjweh.4117

**Published:** 2023-10-01

**Authors:** Lene Aasdahl, Martin Inge Standal, Roger Hagen, Marit Solbjør, Gunnhild Bagøien, Heidi Fossen, Vegard Stolsmo Foldal, Johan Håkon Bjørngaard, Tarjei Rysstad, Margreth Grotle, Roar Johnsen, Egil A Fors

**Affiliations:** 1 Department of Public Health and Nursing, Faculty of Medicine and Health Sciences, Norwegian University of Science and Technology, Trondheim, Norway.; 2 Unicare Helsefort Rehabilitation Centre, Rissa, Norway.; 3 NTNU Social Research, Trondheim, Norway.; 4 Department of Psychology, University of Oslo, Norway.; 5 Department of Psychology, Norwegian University of Science and Technology, Trondheim, Norway.; 6 Research Institute, Modum Psychiatric Center, Vikersund, Norway.; 7 Nidelv Community Mental Health Center, Tiller, Clinic of Mental Health St. Olavs University Hospital, Trondheim, Norway.; 8 The Norwegian Labor and Welfare Service of Trøndelag, Trondheim, Norway.; 9 Norwegian Labour Inspection Authority, Trondheim, Norway.; 10 Faculty of Nursing and Health Sciences, Nord University, Levanger, Norway; 11 Department of Rehabilitation Science and Health Technology, Faculty of Health Sciences, Oslo Metropolitan University, Oslo, Norway.; 12 Centre for Intelligent Musculoskeletal Health, Department of Rehabilitation and Health Technology, Oslo Metropolitan University, Oslo, Norway.; 13 Department of Research and Innovation, Division of Clinical Neuroscience, Oslo University Hospital, Norway.

**Keywords:** case management, mental health, musculoskeletal disease, occupational health, return to work

## Abstract

**Objective:**

This study aimed to evaluate the effectiveness of motivational interviewing (MI) – a counselling approach offered by caseworkers at the Norwegian Labor and Welfare Administration (NAV) – on return to work (RTW) for individuals sick-listed for ≥8 weeks due to any diagnoses. MI was compared to usual case management and an active control during 12 months of follow-up.

**Methods:**

In a randomized clinical trial with three parallel arms, participants were randomized to MI (N=257), usual case management (N=266), or an active control group (N=252). MI consisted of two MI sessions while the active control involved two sessions without MI, both were offered in addition to usual case management. The primary outcome was number of sickness absence days based on registry data. Secondary outcomes included time to sustainable RTW, defined as four consecutive weeks without medical benefits.

**Results:**

The median number of sickness absence days for the MI group was 73 days [interquartile range (IQR) 31–147], 76 days (35–134) for usual care, and 75 days (34–155) for active control. In total 89%, 88% and 86% of the participants, respectively, achieved sustainable RTW. The adjusted hazard ratio (HR) for time to sustainable RTW was 1.12 (95% CI 0.90–1.40) for MI compared to usual case management and HR 1.16 (95% CI 0.93–1.44) compared to the active control.

**Conclusions:**

This study did not provide evidence that MI offered by NAV caseworkers to sick-listed individuals was more effective on RTW than usual case management or an active control. Providing MI in this context could be challenging as only half of the MI group received the intervention.

Long-term sickness absence is a challenge in most western countries, with negative consequences for both the individual and society (1, 2). Return to work (RTW) after long-term sick leave is a complex process where medical, psychological, and social factors are of importance (3, 4). Several stakeholders are often involved in the process, including the employer, the social insurance office, the general practitioner, and other healthcare workers (3, 5). Navigating this process can be challenging, and the sick listed individual may struggle with feelings of poor self-efficacy and motivation to make the necessary changes to achieve a successful RTW (6).

There has been considerable research on RTW interventions, but with inconclusive results (7–11). Early interventions have been advocated to avoid long-term sick leave and permanent exclusion from the labor market (3, 12). However, as most sick-listed workers return to work in less than a month (13, 14), early complex interventions might delay the process (15). Consequently, a stepped-care approach where you start with low-intensity interventions and progress to more complex interventions when the lower interventions fail has been suggested (16, 17).

Motivational interviewing (MI) can be offered as a low-intensity intervention. MI is a counselling approach that has been suggested effective for behavioral change even after only a few sessions (18–20). MI is person-centered, and the counsellor guides the individual to strengthen the person's own motivation and commitment to change (21). MI has been suggested to be useful in the RTW process, and while the literature is sparse, there have been some promising results (22, 23). In Norway at the Norwegian Labour and Welfare Administration (NAV) caseworkers play a central role in the follow-up of sick-listed workers by coordinating RTW efforts. A recent study found a small, but promising, effect of MI on sick leave when offered by NAV caseworkers for individuals sick-listed due to musculoskeletal disorders (22). However, the study only had six months of follow-up and only targeted individuals with musculoskeletal diagnoses. Previous research on RTW interventions has often focused on specific diagnoses (7, 10). However, there is substantial overlap in symptoms and prognostic factors among sick-listed individuals (24, 25), and RTW is therefore argued to be a general process (26). Interventions that can be offered more broadly will also have the potential to reach more people. Research on interventions targeting individuals regardless of their diagnosis is thus warranted. The present study aimed to evaluate the effectiveness of adding two sessions of MI to usual case management offered by NAV caseworkers for individuals sick-listed due to any diagnoses, compared to usual case management alone and an active control arm on sickness absence during 12 months of follow-up.

## Methods

### Study design

We conducted a randomized clinical trial with three parallel arms. The trial compared MI to usual case management and active control for sick-listed individuals. The primary outcome was number of sickness absence days during 12 months of follow-up. The protocol has been published (27) and the study is registered at ClinicalTrials.gov (no. NCT03212118). The Regional Committees for Medical and Health Research Ethics in South East Norway (No: 2016/2300) approved the study, and the results are presented according to the CONSORT statement (28).

### Study context

In Norway, all legal residents are included in the Norwegian public insurance system. Medically certified sick leave is compensated with 100% coverage for the first 12 months if needed, subject to certain salary limitations. The employer covers the first 16 days, while the rest is covered by the Norwegian Welfare and Labor Administration. Graded sick leave (≥20%) is encouraged when possible. Within four weeks of sick leave, the employer and the sick-listed worker must create a plan for RTW. The employer is responsible for arranging a dialogue meeting with the sick-listed worker within seven weeks of absence; other stakeholders may attend when relevant. NAV caseworkers have a counseling role in sickness absence follow-up by providing support for the employer and sick-listed worker, but they also act as a controller of eligibility for sickness benefits. However, they do not meet the sick-listed individuals routinely before a secondary dialogue meeting held around (at the latest) 26 weeks of sick leave. This meeting is only held for those who are 100% sick-listed (ie, not for graded sick leave), and they are only held if NAV caseworkers deem it necessary. However, individuals on sick leave can reach out to their caseworker for assistance or to schedule a meeting through a secure online portal. After 12 months of sick leave, it is possible to apply for more long-term medical benefits: work assessment allowance and disability pension, which both cover approximately 66% of the income.

### Participants

Potential participants were individuals aged 18–60 years, living in Trondheim, and registered with the NAV office participating in the project. Participants had to be on sick leave for ≥8 weeks with a current sick leave status of 50–100% and speak Norwegian. They were excluded from the study if their sick leave was due to pregnancy-related reasons or if they did not have an employer, such as being unemployed or self-employed.

### Recruitment, randomization, and blinding

Potential participants were identified by NAV and contacted through their secure electronic communication website. They received information about the project and accepted or declined participation through the same website. They were informed that whether they chose to participate or not, it would not affect their medical benefits in any way. A NAV employee, who also was a member of the project, verified eligibility for individuals who accepted to participate in the study and sent lists of participants to the researchers for randomization.

Eligible individuals who signed consent were randomized to one of the three groups: MI, active control, or usual case management. To make sure the randomization was concealed for the researchers and participants, block randomization with unknown sizes was carried out using a web-based program provided by a third party, specifically the Unit of Applied Clinical Research at the Norwegian University of Science and Technology (NTNU). The researchers were blinded for group affiliation until all main analyses were performed and interpreted. It was not possible to blind participants or the NAV caseworkers offering the intervention.

### Interventions

*Usual case management.* The usual case management group received standard follow-up by NAV as described in study context. The MI and the active control group received the standard follow-up in addition to the interventions.

*The MI intervention.* The MI intervention consisted of two face-to-face sessions with the caseworker at 7 and 9 weeks after inclusion (ie, 14 and 16 weeks of sick leave). Each session lasted a maximum of 60 minutes. The sessions were based on manuals developed by two of the researchers [a psychiatrist (GB) and a psychologist (RH)], both experienced MI trainers, to ensure that the sessions consisted of valid MI content (the MI manual is available in the online supplementary material, www.sjweh.fi/article/4117). The first session aimed to engage the sick-listed worker in a collaborative relationship with the caseworker and included agenda mapping as well as evoking the person’s motivations for RTW. There was also an assessment of where the sick-listed worker was according to the stages of change model (29), in order to be able to adjust the intervention accordingly. In the second session, the aim was to map the sick-listed individual’s work tasks, earlier attempts of RTW, and RTW self-efficacy and to assess their readiness for RTW. There was also information exchange about possible support from NAV to help RTW. If the worker was ready for change after the MI session, they created a written action plan for RTW together with their caseworker.

The caseworkers who offered the MI intervention underwent comprehensive training offered by the project's MI trainers. Selection of the caseworkers were based on their expressed interest in participating in the project. Many of the caseworkers in the project had undergone some MI training before the project started, and those without previous training were given 3×2 days of workshops in MI. To assure a certain level of MI skills, the caseworkers audio- or video-recorded a role-play using the MI guideline after the initial training, with feedback on MI microskills. An independent Motivational Interviewing Treatment Integrity (MITI) lab evaluated a random selection of 20 audio-recordings (30). During the inclusion period, the caseworkers received 90 minutes of supervision from the MI specialists biweekly.

*Active control.* The active control group received two sessions without MI content, offered by a caseworker. Similarly, as with the intervention group, the sessions were held at 7 and 9 weeks after inclusion and lasted a maximum of 60 minutes each. A guideline for the sessions was developed by the researchers with a focus on general discussions about RTW trying to mimic topics caseworkers usually talk about with sick-listed workers. This intervention arm was designed to control for attention bias from the MI intervention. Previous studies have shown that even minimal interventions impact RTW (31). To accurately assess the effect of MI, and not only caseworkers having a conversation with the sick-listed workers, the active control was designed so that it was identical to the MI intervention with caseworkers offering sessions at the same point in time. The only difference was that the active control did not include MI.

### Outcome measures

The primary outcome was the number of sickness absence days during a 12-month period following randomization. Secondary work outcomes were time until sustainable RTW, which was defined as a period of four consecutive weeks without any benefits (sick leave payments, work assessment allowance or disability pension), and probability of not receiving benefits each month during follow-up. For participants with a graded disability benefit at inclusion any increase in disability pension during follow-up was counted as sick leave. Sick leave data was obtained from the Norwegian National Social Security System Registry, where all individuals receiving any form of sickness or disability benefits in Norway are registered by their social security number. Based on information from the different medical benefits (sick-leave payments, work assessment allowance and disability pension), we calculated the equivalent of full workdays on medical benefits according to a 5-day workweek for every month during follow-up. We also used data from the registry on main diagnoses for sick leave and information about emigration and death. The diagnoses are coded according to the International Classification of Primary Care, second edition (ICPC-2),and were collapsed into three groups: musculoskeletal disorders, mental health disorders, and other.

Other variables registered by questionnaires at inclusion were level of education, dichotomized as high (college/university) or low, subjective health evaluation (scored as poor, not so good, good, or very good) and employment percentage (continuous). Employment percentage was measured by a question about how much they used to work before being sick listed, ie, percentage of full-time employment (0–100%).

### Sample size

Sample size calculation was based on number of sickness absence days (primary outcome). Assuming an average of 60 sickness absence days per year for the control group and 50 days for the intervention group [alpha 0.05, standard deviation (SD) 30], we would need 149 participants in each arm for a Wilcoxon rank-sum test with 80% power. As power calculations are heavily based on assumptions, the planned sample size was increased to 250 in each group. The sample size calculation was performed by a statistician outside the project group.

### Statistical analysis

Median number of sick leave days was compared with the Kruskal Wallis test. We estimated time until sustainable RTW (one month without receiving any medical benefits) using Kaplan Meier curves and log rank test. Hazard ratios (HR) were estimated using Cox proportional model with Efron method for ties. Time was calculated as number of days and participants were censored when they achieved sustainable RTW, death, or at the end of follow-up. Analyses were performed unadjusted and adjusted for age (continuous), gender, education, main diagnosis for sick leave and length and type (full/partial) of sick leave at inclusion. Probability of not receiving any medical benefits each month during follow-up was analyzed as repeating events with logistic General Estimating Equations (GEE). We used an exchangeable correlation structure and without robust standard errors. The analyses were performed unadjusted and adjusted (with the beforementioned variables). All analyses were performed in line with the intention-to-treat principle. In, addition we calculated an estimated per-protocol effect for MI compared to usual case management, adjusting for possible confounding factors of compliance (age, gender, education, and diagnosis).

Precision was assessed using 95% confidence intervals (CI). The statistical analysis plan (SAP) was published at ClinicalTrials.gov (no. NCT03212118) prior to the completion of inclusion (ie, before analyses were started). All analyses were done using STATA 17.0 (StataCorp LLC, Stata Statistical Software: release 17. College Station, TX, USA).

## Results

**Figure 1:**
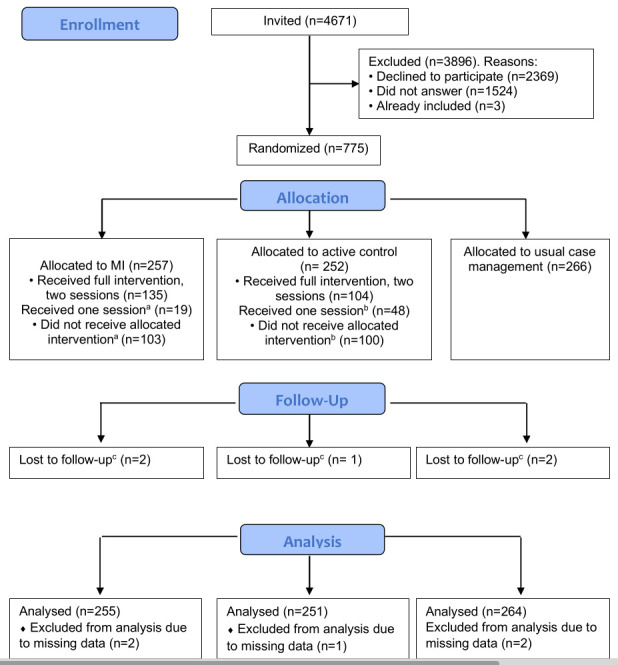
Flow of participants through the study. ^a^ Reasons for not receiving MI intervention: already returned to work=20, illness=6, no time/not possible to manage=4, pregnant=3, language problems=2, no appointment made by case worker =4, other=4, unknown=60. Reasons for only receiving one MI session: already returned to work=3, illness=1, no appointment made by case worker =1, unknown=14. ^b^ Reasons for not receiving active control: already returned to work=18, Illness=5, no time/not possible to manage=6, no appointment made by case worker =5, pregnant= 3, language problems=3, other=4, unknown=56. Reasons for only receiving one session: Already returned to work=17, Illness=1, no time/not possible to manage=1, other=5, unknown=24. ^c^ Due to miscommunication, registry data was not obtained on five participants.

Between January 2018 and October 2020, a total of 4666 individuals were invited to the study, 773 accepted the invitation and 770 were randomized to MI (N=257), usual case management (N=266) and active control group (N=252). Due to a miscommunication, registry data was not obtained on five participants (MI=2; usual case management=2; active control=1), and they were excluded from the analyses. The flow of participants in the study is illustrated in figure 1. Table 1 presents participant characteristics. The mean age was 44 (SD 10) years, and the majority were women (62%). The dominating diagnoses for sick leave were musculoskeletal disorders (42%) and mental health disorders (30%), followed by neurological (8%), general and unspecified (4%), digestive (2%) and respiratory disorders (2%). The rest was spread across the remaining diagnoses groups (12%).

**Table 1:** Baseline characteristics for the included participants (N=770).^a^ [SD=standard deviation; IQR=interquartile range.]

		Motivational interviewing (N=255)			Usual case management (N=264)			Active control (N=251)	
		% (N)	Mean/median (SD/IQR)		% (N)	Mean/median (SD/IQR)		% (N)	Mean/median (SD/IQR)
Age			44 (10)			44 (10)			44 (10)
Women		63 (160)			61 (161)			63 (157)	
Higher education ^b^		45 (116)			51 (135)			49 (123)	
Employment status									
	Full time	73 (185)			77 (202)			71 (179)	
	Part time	24 (63)			20 (53)			24 (60)	
	Graded disability pension ^c^	3 (7)			3 (9)			5 (12)	
Sick-leave status ^d^									
	Full-time sick-leave	49 (125)			50 (131)			44 (111)	
	Partial sick-leave	50 (128)			50 (131)			55 (139)	
	Work assessment allowance	0.8 (2)			0			0	
	No benefit	0			0.8 (2)			0.4 (1)	
Main diagnoses for sick-leave (ICPC-2) ^d^									
	L- musculoskeletal	41 (105)			43 (114)			41 (103)	
	P- psychological	31 (78)			31 (82)			28 (71)	
	Other	28 (72)			26 (68)			31 (77)	
	Sick leave days the year before inclusion ^e^		78 (65–97)			77 (67–101)			77 (68–96)
Type of work ^f^									
	Mostly sedentary	32 (82)			34 (90)			34 (85)	
	Much walking	12 (31)			18 (47)			13 (32)	
	Much walking and lifting	20 (51)			21 (56)			24 (60)	
	Heavy physical work	5 (14)			3 (9)			5 (12)	
	Not sure/missing	30 (77)			23 (62)			25 (62)	
Subjective health evaluation									
	Poor	13 (33)			14 (37)			17 (42)	
	Not so good	38 (97)			46 (121)			43 (108)	
	Good	19 (49)			17 (44)			14 (34)	
	Very good	1 (2)			1 (3)			2 (6)	
	No response	29 (74)			22 (59)			24 (61)	

^a^ Due to a miscommunication registry data was not obtained on five participants and excluded from the analyses [motivational interviewing (N=2); usual care (N=2); active control (N=1)]. ^b^ Higher (tertiary) education (college or university); missing data on N=68, 53 and 56 for the respective groups. ^c^ Individuals working part time that at inclusion also received a graded disability pension. ^d^ Based on data in the medical certificate from the National Social Security System Registry. ^e^ Number of days on sick leave during the last 12 months prior to inclusion. Measured as calendar days, not adjusted for graded sick- leave or part time job. ^f^ Based on the self-reported data to the question “How would you describe your work?”

### Sickness absence days

**Figure 2:**
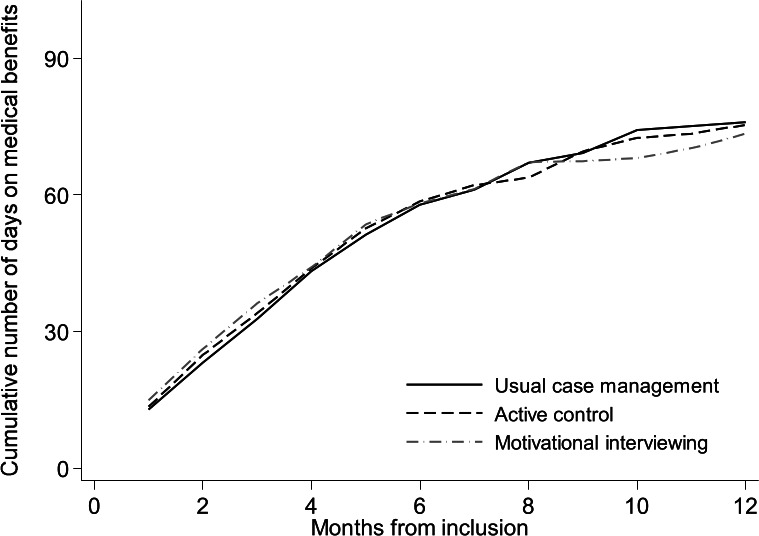
Cumulative number of workdays (median) on medical benefits for the motivational interviewing, usual case management and active control group during 12 months of follow-up, based on intention to treat analyses. Number of days are adjusted for employment fraction and transformed to whole workdays according to a 5-day workweek

The median number of sick leave days was lowest for the MI group with 73 [interquartile range (IQR) 31–147] days. For the other two groups the median was 76 (IQR 35–134) days for the usual case management and 75 (IQR 34–155) days for the active control group. The difference between the groups was not statistically significant (Kruskal Wallis P=0.773). Figure 2 shows the cumulative median number of sickness absence days during 12 months of follow-up.

### Return to work

**Figure 3:**
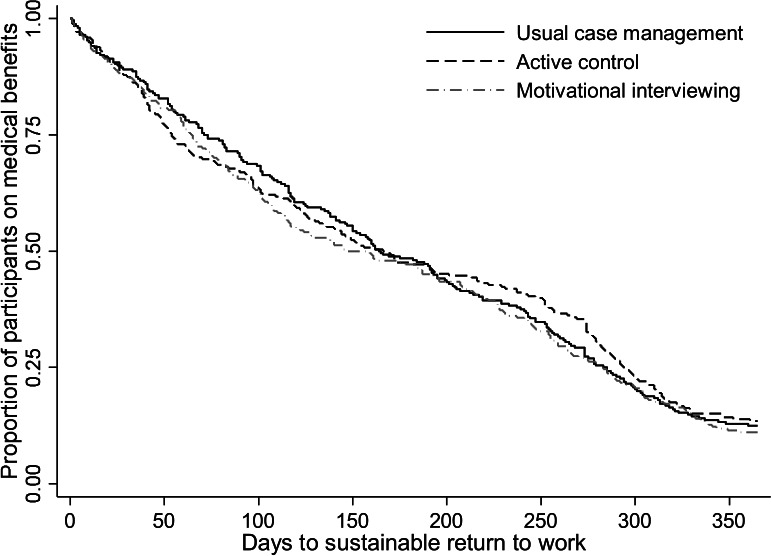
Survival curves from the Kaplan Meier intention to treat analysis showing time to sustainable return to work (ie, 1 month not receiving medical benefits) for the motivational interviewing, usual case management and active control group during 12 months of follow-up.

During 12 months of follow up 89%, 88% and 86% of the participants achieved sustainable RTW in the MI, usual case management, and active control group, respectively. One participant died during follow- up and was censored. Two participants emigrated, but this was close to the end of follow-up, and they did not receive medical benefits at the time they emigrated. The Kaplan-Meier plot is shown in figure 3. The difference between the groups was not statistically significant (log rank test P=0.689). Median time to sustainable RTW was 159 days for the MI group, 161 days for the usual case management, and 168 days for the active control group. The unadjusted HR for sustainable RTW was 1.01 (95% CI 0.92–1.11) for the MI group compared to the usual case management and 1.08 (95% 0.90–1.31) compared to the active control group. The adjusted HR were 1.12 (95% CI 0.90–1.40) and 1.16 (95% CI 0.93–1.44), respectively. The estimated per-protocol effect for MI compared to usual case management gave a HR for sustainable RTW of 1.00 (95% CI 0.78–1.29).

Several participants experienced new sick leave episodes during follow-up, and at 12 months the number of participants at work was 65%, 69% and 64% for the MI, usual case management, and active control groups, respectively. When evaluating sustainable RTW during the follow-up period, the group that received MI had a slightly larger probability of RTW for certain months, but the groups were closely matched during the first few months of follow-up (as shown in the supplementary figures S1 and S2). Number of participants transitioning to the more long-term benefit work assessment allowance was lower in the usual case management group (14%) than in the MI (19%) and active control group (21%).

### Compliance

In the MI group, 53% received two MI sessions, 7% one session, and 40% no sessions. For the active control group, the numbers were 41%, 19%, and 40% respectively. Reasons for not receiving the interventions are described in figure 1 when known, however, most are unknown due to participants not giving a reason (due to research ethics not required). There was no clear pattern in compliance increasing or decreasing over time. Participants who received two MI sessions were more likely to be female (67% versus 62%) and have higher education (66% versus 56%) compared to those who received no sessions. The average age was similar between the two groups (mean 44 versus 43). Musculoskeletal disorders dominated for both those receiving two MI sessions and no MI sessions (41% and 43%), while psychological diagnoses were more common for those receiving two sessions than those receiving no MI sessions (35% versus 27%). Those who received two MI sessions had more sick leave during follow-up: median 103 (IQR 49–161) days compared to those with no sessions 45 (IQR 20–112) days.

## Discussion

Our study found weak or no support of any MI effect compared to usual case management or an active control group in terms of future work participation. Although the MI and usual case management group did slightly better than the active control group over time, the groups were closely matched during the first months, making it unlikely that these small differences were due to the intervention. However, only about half of the participants showed up for the MI intervention.

Our findings differ from previous studies that have found results indicating a positive effect of MI on RTW, albeit the effects have been small with low precision (22, 23, 32). One difference between the present study and a recent Norwegian study (22) which found a small, but promising, effect of MI offered by NAV caseworkers for RTW, was compliance to the intervention. The low compliance in the present study means the results should be interpreted with caution. Another difference was the target group, where the previous study included only musculoskeletal disorders, while we included unselected sick-listed individuals. The wide inclusion criteria resulted in inclusion of participants who were unable to RTW due to severe illnesses or waiting for operations. However, a recent pilot study in Belgium (32) that included all diagnoses, found that offering a single short consultation (15–20 minutes) of MI in a social security setting led to faster RTW and a longer time before relapse. In contrast to our study, MI in the Belgian study was offered by a psychologist with a certification in MI and not by caseworkers. While elements of MI can be useful communication tools, previous studies have shown that to gain competence in MI one needs practicing and sufficient time to learn it properly (33–35). In a process evaluation alongside the current study, the caseworkers expressed that they did not have enough time in their schedule to practice their MI skills and that it was difficult to use in conversations with the sick-listed workers, which was reflected in fidelity (MITI) scores that were varying and some below average (36). The caseworkers reported that some MI skills were more challenging to learn than others. This is in line with other studies trying to implement MI in a social insurance setting (33, 34). MI has traditionally been used in clinical settings and it might be easier for clinicians than NAV caseworkers to learn MI to the level needed. It has also been suggested that MI might be more effective when combined with another treatment, such as cognitive behavior therapy or occupational rehabilitation (32, 37). In a qualitative study on the experiences of receiving the MI intervention, the participants described a good and positive relationship with the MI caseworkers (38). However, the dual role of the caseworker as both counsellor in sickness absence follow-up and controller of eligibility for sickness benefits is a challenge in the social insurance setting and may affect the sick listed worker’s trust in the caseworker (39). Furthermore, in social security settings it varies how often caseworkers can practice their MI skills and hence improve them over time (33, 36). It is therefore also possible that there was too little room for practicing MI, reflecting that implementation requires that the organization to a more extensive degree must provide suitable time and workload for the caseworker.

The MI intervention included only one stakeholder, the social security office. Due to the complex nature of sick leave, it has been suggested that involvement, coordination, and communication between the different stakeholders are important (3, 7, 40). In this study, there was no communication with other stakeholders beyond what is part of usual case management. A potential explanation for the lack of effect of the intervention could be the lack of coordination with the participants' general practitioner, as they are the ones that issue sick leave notes in Norway. Given that the general practitioner knows the sick-listed worker over time, has clinical training and writes sick leave notes, future studies should evaluate the effect of general practitioners’ using MI in consultations with sick-listed patients. Furthermore, there is a need for more communication between the stakeholders. Many sick-listed workers experience a distance to the NAV office, where the contact is mainly standardized letters about their duties and rights (41). In qualitative studies from this project, both sick-listed workers and the NAV caseworkers reported positive experiences with the MI intervention (36, 38). However, the sessions were not perceived as beneficial if there already was a clear plan for RTW. This suggests that MI may be more useful when targeted to specific groups of individuals, and more research is therefore needed to determine in which settings and for whom MI is an effective intervention.

Our trial has several strengths: the use of registry data resulted in almost no missing data on the main outcome, the use of an active control group helped to eliminate potential attention bias, the intervention was designed to achieve a biopsychosocial approach to sick leave, and recruitment through NAV ensured no referral bias. The main limitation of our trial was that many participants did not show up for the sessions at the NAV office. This could be due to the inclusion of participants with different diagnoses, including severe illnesses like cancer, but could also be due to the delay between randomization and the intervention, which resulted in many returning to work before the intervention took place. This is also a likely explanation for why participants not showing up for the MI sessions had fewer sickness absence days than those receiving the two sessions. Contamination between the arms is a risk since MI is a tool NAV wants its caseworkers to use. However, the fact that MI may be hard to master might have reduced the risk. In this study, we used the absence of medical benefits as an indicator of being at work. In Norway, employees who are absent from work due to illness are protected from dismissal for the first 12 months. This, combined with a high employment rate for both men and women and a generous insurance system, makes the absence of medical benefits a fair proxy for having returned to work. However, it is important to acknowledge that some individuals may have transitioned to other welfare benefits or received support through by private means. It should also be noted that the last part of the trial took place during the COVID-19 pandemic, this affected the intervention for about 15–20 participants in each group who received the interventions digitally. Furthermore, a large part of the participants, although having completed the intervention, were still enrolled in the trial during the pandemic. It is not possible to know how that affected their sick leave, but there is no reason to believe it affected the groups differently.

### Concluding remarks

This study did not provide evidence that MI was more effective than usual case management or an active control on RTW. Based on these results and the fact that only half of the participants attended the intervention, it implies that NAV may not be the ideal setting for this type of intervention among this particular group of unselected patients.

### Ethical approval

All procedures performed in studies involving human participants were in accordance with the ethical standards of the institutional and/or national research committee and with the 1964 Helsinki Declaration and its later amendments or comparable ethical standards. The Regional Committee for Medical and Health Research Ethics in South East Norway approved the study (No: 2016/2300).

### Consent

Written informed consent was obtained from all individual participants included in the study. No consent was needed for publication.

## Supplementary material

Supplementary material

## Data Availability

Data is not available due to ethical approval.
