# Supplementary material for: Effectiveness of 'motivational interviewing' on sick leave: a randomized controlled trial in a social insurance setting
Source: Scand J Work Environ Health. 2023 Sep 29;49(7):477–86. doi: 10.5271/sjweh.4117 (PMC10834143; doi:10.5271/sjweh.4117)
Supplement: Supplementary material [file SJWEH-49-477-S001.pdf]

Effectiveness of 'motivational interviewing' on sick leave: a randomized controlled trial in a social insurance setting[1]

by Lene Aasdahl, PhD,[2] Martin Inge Standal, PhD, Roger Hagen, PhD, Marit Solbjør, PhD, Gunnhild Bagøien, PhD, Heidi Fossen, Msc, Vegard Stolsmo Foldal, PhD, Johan Håkon Bjørngaard, PhD, Tarjei Rysstad, PhD, Margreth Grotle, PhD, Roar Johnsen, PhD,1 Egil A Fors, PhD

1. Supplementary material
2. Correspondence to: Lene Aasdahl, PhD, Department of Public Health and Nursing, NTNU, Faculty of Medicine and Health Sciences, Postboks 8905, 7491 Trondheim, Norway.[E-mail: lene.aasdahl@ntnu.no]

MI manual based on the main themes

Session 1:

- Introduction of session 1 from the interviewer – the frame of the conversation
- Agenda mapping: the sick listed person (SLP) chooses theme(s) from a sheet for agenda mapping (e.g family, mental health, economy, etc.)
- The interviewer adapt the intervention to the sick-listed`s stage of motivation for RTW
- Rating scale about the SLP`s importance of being able to work and follow-up questions
- Rating scale about the SLP `s confidence in being able to work and follow-up questions
- The interviewer provides a summary of session 1 to the SLP

Session 2:

- Introduction and summary of session 1 (previous conversation)
- Introduction to session 2 from the interviewer
- Assess the SLP`s current work situation
- Assess the SLP`s previous and / or possible work attempts
- Information on possible contributions from the Norwegian Labour and Welfare Administration (NAV)
- Rating scale about the SLP`s confidence in being able to work and follow-up questions
- Explore the SLP`s goals and values in life
- Scaling of readiness for RTW and follow- up questions
- Summary of both session 1 and 2 with key questions
- Intervention in the last part of the conversation adapted to the stage of motivation
- Thanking the participant for the conversation

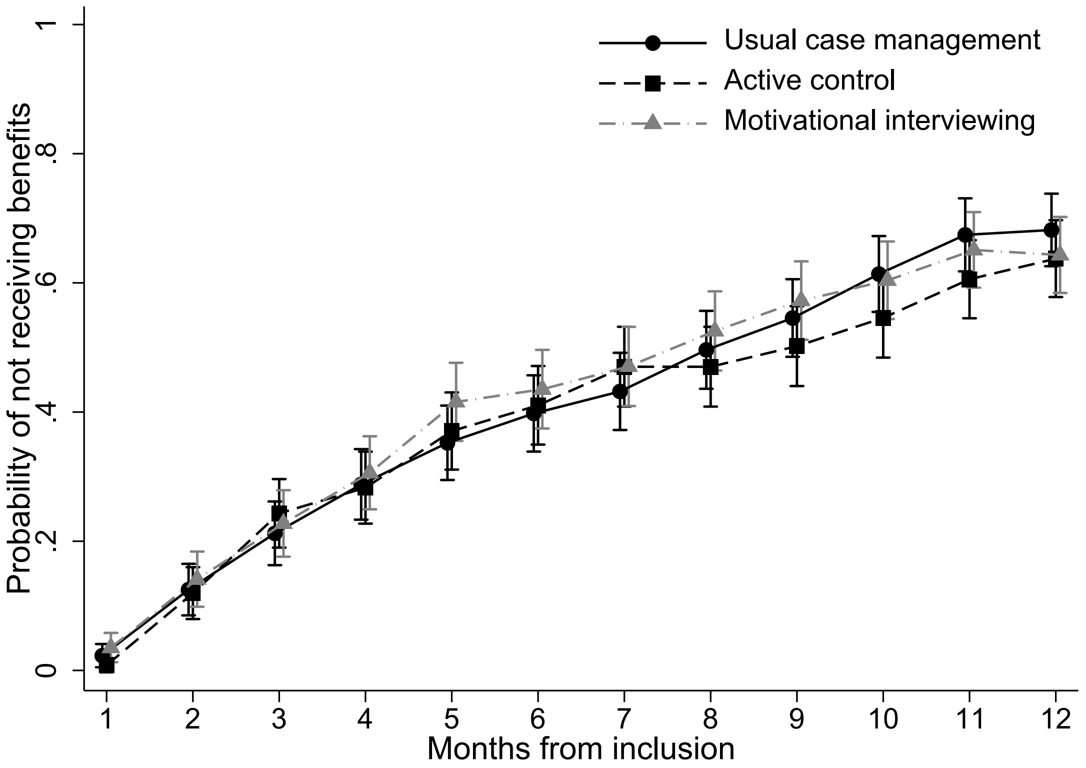

Online supplementary figure 1 Estimated probability of not receiving medical benefits for each month during follow-up for the motivational interviewing, usual case management, and active control group based on logistic General Estimating Equations (GEE) analyses.

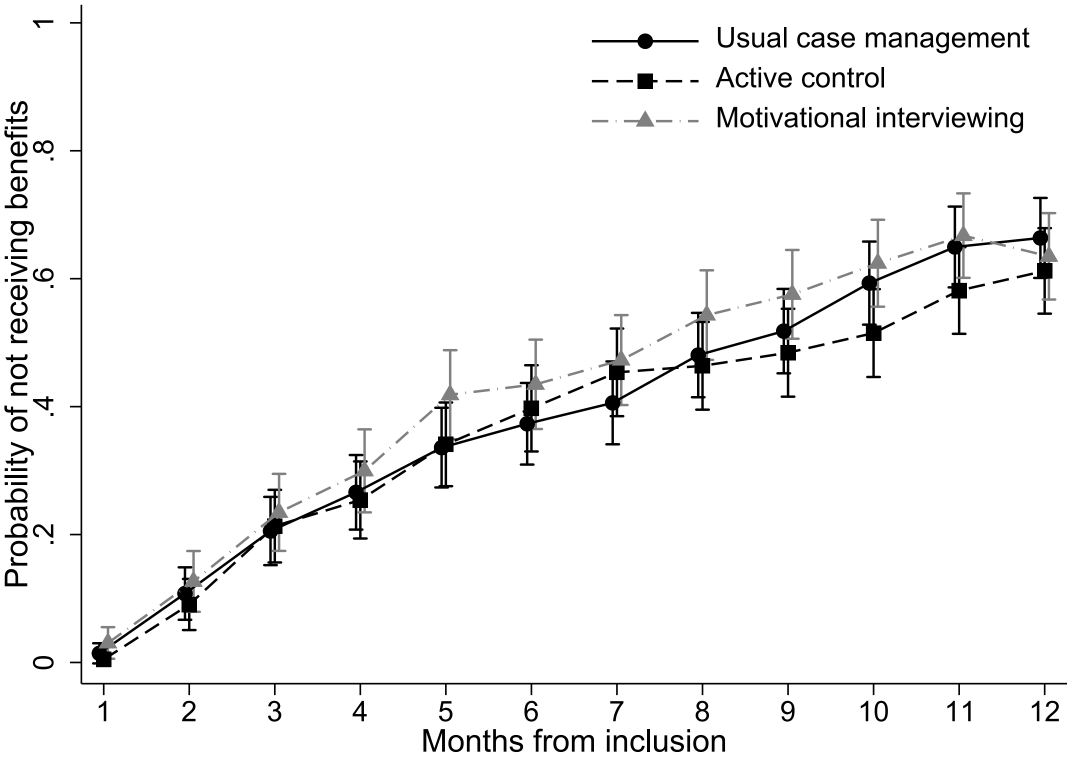

Online supplementary figure 2 Estimated probability of not receiving medical benefits for each month during follow-up for the motivational interviewing, usual case management, and active control group. Logistic General Estimating Equations (GEE) adjusted for age, gender, education, main diagnosis and length and type (full/partial) of sick leave at inclusion.
